# Supplementary material for: Visualization of Three Sclerotiniaceae Species Pathogenic on Onion Reveals Distinct Biology and Infection Strategies
Source: Int J Mol Sci. 2021 Feb 13;22(4):1865. doi: 10.3390/ijms22041865 (PMC7918164; doi:10.3390/ijms22041865)
Supplement: Supplementary file 1 [file ijms-22-01865-s001.pdf]

International Journal of
Molecular Sciences

MDPI

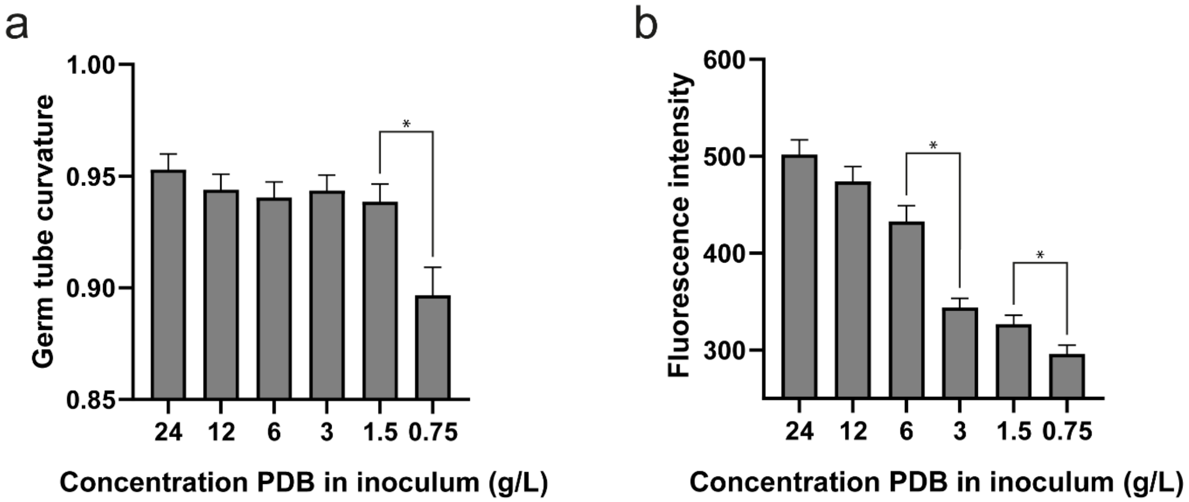

Figure S1. The effect of nutrient concentration in the inoculum on (**a)** curvature and (**b**) intensity of fluorescence of germ tubes of *B. squamosa* at 8 HPI on the onion leaf surface. Error bars representing standard error, unpaired *t*-test, * $p<0.05$.

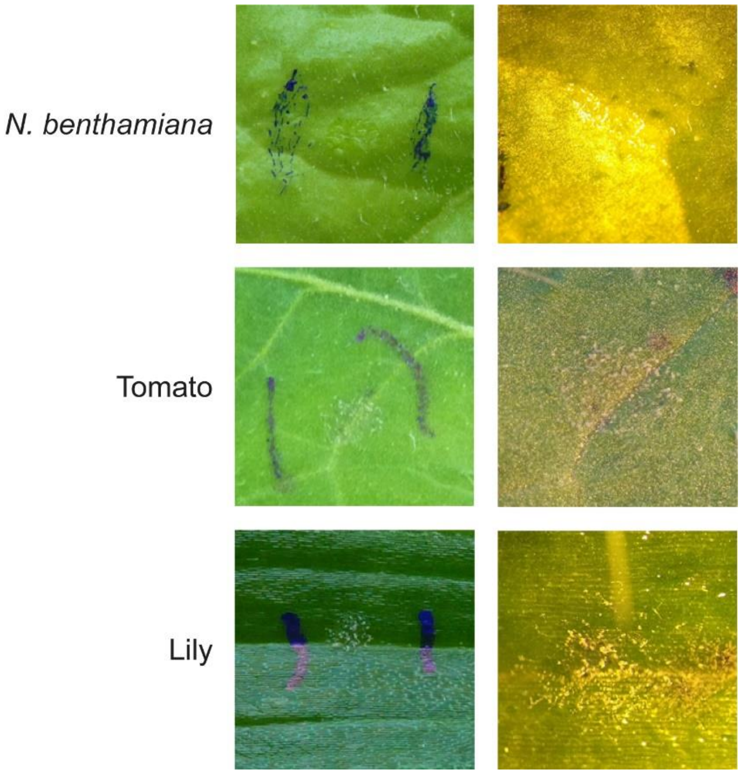

Figure S2. Different plant species inoculated with *B. aclada* at 72 HPI. Not only onion but also non-host plants tomato and lily show sporulation.

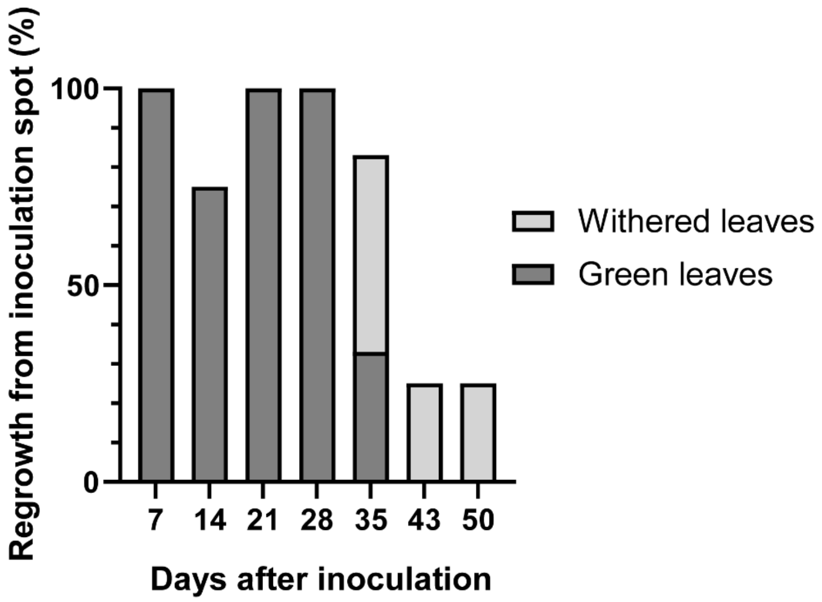

Figure S3. *B. aclada* long term infection assay. Regrowth from inoculated leaf segments over time.
